# Homeopathic Sanitation

**Published:** 1892-10

**Authors:** 


### HOMEOPATHIC SANITATION.

Recently, during the cholera scare, when the people all over the United States were exercised on the subject of cholera invasion, and preventive measures were being discussed everywhere, the good people of San Antonio called upon their county physician for advice.

Now, San Antonio is a remarkable city in many respects. It is a historical city; and around its ancient walls cluster memories of deeds of daring and desperate men; of pure patriotism and chivalry, worthy of commemoration in song and verse. It is remarkable too, for her divided delegations of patriot politicians, for her Paschalls and her Schrams, (as well as for her Sc(h)ram—bles for office); remarkable too for her weird and winding little ways and water courses, (not to mention the devious ways of wayward citizens), and remarkable too is she, not last nor least, for the excellence of her far-famed tamales.

But in addition to all these, San Antonio possesses the enviable distinction of being, so far as this scribe knows, the only city in the world that is blessed with a homeopathic health officer. Yes, by some strange and inscrutable workings, some freak perhaps, of fortune, it happens that *the* city in Texas of all others which one would think likely perhaps, from many causes, to have cholera, should it appear in this section, is blessed with a county physician of the homeopathic persuasion! Why not? some one will ask. Well, if our readers do not see the absurdity of the thing 'twere folly to try to make them understand why not; we will not attempt it.

But a paper published elsewhere in this issue, entitled "Arsenic vs. Cholera," will serve for an answer "why not" appoint a homeopath to so responsible a position; a position where sound knowledge of sanitation, as well as of the etiology of epidemic diseases, and a knowledge of the principles of rational medicine are necessary, and should guide such an officer, instead of a vague delusory and irrational theory of *similars*.

It will be seen by reading the paper referred to, that the author, a representative homeopath, enlightened beyond the time or capacity or pretentions of Mr. Hahnemann, their patron saint, the homeopathic Hippocrates and Galen combined, has advanced homeopathy beyond the limits of Hahnemannism and into the domain of preventive medicine. He goes Mr. Hahnemann one better, by promulgating the doctrine that "like" will not only *cure* "like," but will *prevent* it.

Accepting the homeopathic doctrine thus improved, is it not clear that under the administration of a wise, ambitious and progressive county health officer (and we know Dr. Clifford to be an intelligent and well meaning young man, unfortunately educated in the wrong lines) should not the citizens of San Antonio be taught in accordance with this, the most advanced principle of homeopathy—that, to prevent cholera,—filth and crowds and sour krout and beer and other unhygienic things generally accounted factors in its production, are to be employed *secundem artem?* Or, should a case occur, a case of real cholera, before Dr. Clifford has been enabled to put in operation his own brilliant schemes for keeping it out, would not the proper treatment, homeopathically be, for instance, *arsenic?* as suggested by the writer referred to, or say, tartar emetic, or some other drug or substance which, when administered in large quantities will produce rice water discharges, cramps and collapse? Surely; or even sour krout or beer, or cucumbers; these are the "similars," and according to Dr. Leach, will, or should, not only cure, but prevent cholera.

But our Bexar county health officer had not at that time, heard of this new Hahnemann star risen in the west, nor imbibed the principles of his brilliant theory, and is in a measure excusable for having recommended as prophylaxis, amongst others, a thing as antiquated as cleaning up. Nevertheless, if Dr. Leach *is* a whale, homeopathically; if he has startled the world with a discovery of dazzling brilliancy, Dr. Clifford is no sardine; he has ideas too, not laid down in the book.

In his report to the city council of San Antonio during the interview which was published in the *Light*, along with other suggestions he recommended the following:

PREVENTIVES.

"Should cholera make its appearance I would advise, besides following the conditions set forth in the foregoing, that each individual wear a belt with two or three copper plates about the size of a silver dollar around the abdomen and covered by a flannel band, and that they take the ordinary quill toothpick, fill it with camphor and keep it in the mouth."

The copper-disc is, itself a brilliant disc-overy; or, was it suggested by the story of the stork and the eel,* which doubtless gave the Doctor a vague idea of getting the deadwood on the

*A Stork swallowed a Live Eel, but being a Slippery Fellow, the Eel readily escaped by the Back Way; whereupon the Stork seeing the Game he was Up To, again swallowed the Eel, and backing himself up against a Log, remarked "I am under the impression that I have got the Dead Wood on you Now."—Æsop.

microbes; of preventing either their entrance into the system, or being in, their exit; if so he failed to designate the proper manner of applying the discs, and stopped *half way*.

But the cholera scare is over; its a back number already, and the gifted young homeopath has retired on his laurels amid the plaudits of an admiring constituency and the benedictions of a ransomed people. *Vive la homeopath!*

